# Overgrowth of mice generated from postovulatory‐aged oocyte spindles

**DOI:** 10.1096/fba.2019-00005

**Published:** 2019-04-15

**Authors:** Kouki Shiina, Masaya Komatsu, Fumi Yokoi, Hanako Bai, Masashi Takahashi, Manabu Kawahara

**Affiliations:** ^1^ Laboratory of Animal Genetics and Reproduction, Research Faculty of Agriculture Hokkaido University Sapporo Japan

**Keywords:** oocyte spindle transfer, postnatal growth, postovulatory aging

## Abstract

Oocyte spindle transfer (OST) is a potent reproductive technology used for mammals that enables the spindle in a deteriorated oocyte at the metaphase of the second meiotic division (MII) to serve as the genetic material for producing descendants. However, whether postnatal growth is achieved via OST using developmentally deteriorated MII oocytes remains unclear. At 16 h after human chorionic gonadotropin administration, denuded MII oocytes immediately after retrieval from oviducts (0 h‐oocytes) were used for in vitro fertilization (IVF) as controls. For IVF using postovulatory‐aged oocytes, the 0 h‐oocytes were further incubated for 12 h and 24 h (12 h‐ and 24 h‐oocytes). These mouse oocytes served as a model for assessing the postnatal growth of individuals produced via OST from developmentally deteriorated oocytes. The embryos from 12 h‐ and 24 h‐oocyte spindles exhibited high rates of development up to the neonatal stage as good as the non‐manipulated controls. However, the mice derived from the 24 h‐oocyte spindles displayed heavier body weights and greater feed consumption than both controls and mice derived from 12 h‐oocyte spindles. Our results demonstrate the feasibility of OST as a potent reproductive technology and its limitation in the use of excessively aged postovulatory oocytes in mammalian reproduction.

**Abbreviations**
ART: assisted reproduction technology
BSA: bovine serum albumin
DMR: differentially methylated region
hCG: human chorionic gonadotropin
HTF: Human tubal fluid
IU: International units
IVF: in vitro fertilization
MII: the metaphase of the second meiotic division
OST: oocyte spindle transfer
PBS: phosphate‐buffered saline
PCR: polymerase chain reaction
PVA: polyvinyl alcohol
SDS: sodium dodecyl sulfate
ZP: zona pellucida

## 1 INTRODUCTION

Oocytes are essential for all types of reproduction in mammals. For normal reproduction through fertilization, including somatic cell animal cloning,^1^ and sperm‐free mouse production,^2^ the preparation of oocytes to support full‐term development is a prerequisite for completing ontogeny. Hence, the developmental competence of an embryo is critically dependent on the soundness of the oocyte, which is deteriorated by various factors, including toxic chemicals,^3^ cryodamage,^4^ and aging of oocyte‐supplying females.^5^ The postovulatory duration is also a well‐known potentially influential factor that causes various serious impairments in mammalian oocytes.^6^, ^7^, ^8^ It is easily conceivable that oocytes are susceptible to postovulatory aging when females miss the timing for optimal fertilization after ovulation.

Oocyte spindle transfer (OST) is an assisted reproduction technology (ART) that is expected to fundamentally reverse ooplasmic deterioration, as tested using mouse oocytes.^9^, ^10^, ^11^, ^12^ In humans, OST has been used to avoid mutated mitochondrial DNA transmission from oocytes.^13^ In OST, the spindle‐chromosome complex in a deteriorated oocyte at the metaphase of the second meiotic division (MII) is transferred into an enucleated good‐quality MII oocyte, and this process is not expected to perturb subsequent fertilization and developmental competence. The resultant ooplasm replacement would be a reliable method for avoiding the loss of developmental competence to term and the transmission of diseased mitochondria to descendants. However, the safety of OST has not been fully explored in a mammalian model because the endpoint of evaluations using mouse oocytes in most studies was limited to the gestational full‐term development competence of embryos derived from OST‐oocytes.^9^, ^10^, ^11^ To build a consensus that OST is a safe ART for human reproduction, it is essential to ensure the normal growth of individuals after birth in an animal model.

Without assessments of postnatal growth, it is impossible to argue for the application of OST as a practical reproductive technology. Therefore, we investigated both development at birth and conducted postnatal estimation of growth and feed consumption using a mouse deterioration model of postovulatory‐aged oocytes because there are remarkable cytological similarities regarding the mechanisms of postovulatory aging and other causes of oocyte deterioration, including in vivo aging and cryopreservation.^11^, ^14^, ^15^, ^16^, ^17^, ^18^ Oocyte aging resulting from missing the optimal timing of fertilization could easily be mimicked by postovulatory aging under in vitro culture conditions.^9^, ^11^ In this study, we addressed the postnatal development and growth of OST mice produced by MII spindles from oocytes incubated for 12 or 24 h after collection from females that were administered human chorionic gonadotropin (hCG) to induce ovulation.

## 2 MATERIALS AND METHODS

All research protocols were approved by the Regulatory Committee for the Care and Use of Laboratory Animals, Hokkaido University.

### 2.1 Preparation of mouse oocytes

ICR (CD1) female mice were used as oocyte donors and superovulated via treatment with 7.5 International Unit (IU) of equine chorionic gonadotropin (ASKA Pharmaceutical, Tokyo, Japan) and 7.5 IU of hCG (ASKA Pharmaceutical) administered 48 h apart. At 16 h after hCG administration, cumulus oocyte complexes were collected from oviducts and treated in M2 medium^19^ containing 0.13% hyaluronidase (Sigma‐Aldrich, St. Louis, MO) to remove cumulus cells. After washing in hyaluronidase‐free M2 medium, the denuded MII oocytes were used for in vitro fertilization (IVF) immediately after retrieval from oviducts (0 h‐oocytes). IVF was performed by modifying the methods described in previous studies.^19^, ^20^ For IVF using postovulatory‐aged oocytes, the 0 h‐oocytes were further incubated in M16 medium^21^ in a humidified atmosphere of 5% CO_2_ at 37°C for 12 or 24 h (12 h‐ and 24 h‐oocytes, respectively).

### 2.2 Cortical granule staining in mouse oocytes

Cortical granule (CG) lectin was stained as described previously.^22^, ^23^ Oocytes were fixed with 4% paraformaldehyde (Wako Pure Chemical Industries, Ltd, Osaka, Japan) in Dulbecco's phosphate‐buffered saline (PBS) for 1 h at room temperature, followed by incubation in blocking solution, which comprised PBS containing 0.2% polyvinyl alcohol (PVA; Sigma‐Aldrich), 0.3% bovine serum albumin (BSA) (Sigma‐Aldrich), and 100 mM glycine (Kanto Chemical, Tokyo, Japan) for 5 min. Subsequently, oocytes were washed with PBS/PVA containing 0.1% Triton X‐100 (Wako Pure Chemical Industries, Ltd) and incubated with Alexa Fluor 488‐conjugated lectin PNA (1:200; Molecular Probes, OR) for 30 min at room temperature. After washing in PBS/PVA containing 0.01% Triton X‐100 and 0.3% BSA, oocytes were mounted onto glass slides using VECTASHIELD Mounting Medium (Funakoshi, Tokyo, Japan). Images were obtained using a TCS SP5 confocal laser microscope (Leica, Tokyo, Japan).

### 2.3 In vitro fertilization after OST

OST was performed as previously described with a slight modification.^20^, ^24^ Briefly, the zona pellucida (ZP) around the first polar body of each 0 h‐oocyte was cut using a glass knife in M2 medium supplemented with 7.5 µg/mL cytochalasin B (Sigma‐Aldrich) and 0.1 µg/mL colcemid (Wako Pure Chemical Industries, Ltd, Osaka, Japan). Following the removal of the polar body, the spindle, including a small amount of cytoplasm, from 12 h‐ and 24 h‐oocytes was fused with enucleated 0 h‐oocytes using inactivated Sendai virus (hemagglutinating virus of Japan; Ishihara Sangyo Kaishya, Ltd). Cell fusion was confirmed after 1 h of culturing in M16 medium. The reconstructed oocytes were used for IVF.

Prior to IVF, spermatozoa were collected from the cauda epididymis of mature ICR and preincubated in the droplets of the human tubal fluid (HTF) medium in a humidified atmosphere containing 5% CO_2_ at 37°C for 1.5 h.^25^ The oocytes produced via reconstruction with 12 h‐ or 24 h‐oocyte spindles were transferred into droplets of the HTF medium. Preincubated spermatozoa were added to the same droplets. The presumptive zygotes were washed in M2 medium and transferred into droplets of M16 medium containing 0.1 mM EDTA (Dojindo Laboratories, Kumamoto, Japan) 6 h after insemination for in vitro culture. At 6 h after insemination, the rates of male and female pronuclear formation were assessed in five types of presumptive zygotes: using 0 h‐oocytes (n = 99), 0 h‐oocytes without zona pellucida (ZP) (n = 43), 24 h‐oocytes with a slit in ZP (n = 55), 24 h‐oocytes without ZP (n = 48), and OST‐oocytes reconstructed from 24 h‐oocyte spindles (n = 114). The IVF embryos were cultured to embryonic day 3.5 (E3.5) in a humidified atmosphere containing 5% CO_2_ at 37°C. The embryos that developed to the blastocyst stage were transferred to the uterine horns of recipient female mice at 2.5 days of pseudopregnancy. The fetuses were born via natural delivery. For the following experiment to assess the methylation status in the differentially methylated region (DMR), autopsies were conducted at E16.5, by which time the mouse fetus has completed major organogenesis. For non‐manipulated IVF controls, fetuses/pups were prepared via embryo transfer of blastocysts produced from 0 h‐oocytes that were collected at 16 h after hCG injection.

### 2.4 Measurements of body weight and food intake

All mice were fed a standard diet ad libitum and housed in a time‐controlled lightening system with a 12‐h:12‐h light:dark cycle (lights on, 7:00 h‐19 h). The temperature and humidity were set at 22°C and 50%, respectively. We started the measurements of body weight and food intake when the pups were weaned at 4 weeks of age and fed individually. Body weight was measured weekly, and food intake was measured daily until the mice reached 10 weeks of age. To unify the number of suckling pups per foster mother, we arranged 10 pups in both the OST‐ and control mouse groups.

### 2.5 Bisulfite sequencing

To isolate genomic DNA, the heart, brain, and liver of each female E16.5 fetus were homogenized using a BioMasher (NIPPI, Tokyo, Japan). Subsequently, mashed samples were lysed in 400 μL of TNE buffer (10 mM Tris‐HCl at pH 7.5, 100 mM NaCl, 1 mM EDTA) containing 20 μL of 10% sodium dodecyl sulfate (SDS) solution (Wako Pure Chemical Industries, Ltd) and 8 μL of 10 mg/mL proteinase K solution (Wako Pure Chemical Industries, Ltd), followed by incubation for 16 h at 37°C. Incubated lysates were purified by phenol/chloroform extraction and ethanol precipitation. Resultant genomic DNAs were resuspended in distilled water. The genomic DNA concentration was quantitated using a Nanodrop (Thermo Fisher Scientific, Wilmington, DE). Genomic DNA was treated with bisulfite reagent using an EZ DNA Methylation‐Gold Kit™ (Zymo Research, CA) according to the manufacturer instructions. Bisulfite‐treated genomic DNA was amplified by nested PCR using Takara Ex Taq Hot Start Version (Takara Bio Inc, Shiga, Japan) for the *Snrpn*‐DMR. The primer sets for bisulfite sequencing and nested polymerase chain reaction (PCR) are presented in Table 1.

**Table 1:** Primer sets and PCR condition for *Snrpn*‐DMR analysis

	PCR	Name	Sequences (5'‐3')	PCR condition	Length
Snrpn	1st	Snrpn‐BSF1	ATTGGTGAGTAATTTTTTGGAGT	94°C for 2 min, (94°C for 30 s, 58°C for 30 s, 72°C for 30 s) × 15 cycles, 72°C for 5 min, 4°C for ∞	260 bp
		Snrpn‐BSR1	ACAAAACTCCTACATCCTAAAA		
DMR	2nd	Snrpn‐BSF1	ATTGGTGAGTAATTTTTTGGAGT	94°C for 2 min, (94°C for 30 s, 58°C for 30 s, 72°C for 30 s) × 25 cycles, 72°C for 5 min, 4°C for ∞	
		Snrpn‐BSR2	CACAAACCACACAATTAATAATTCC		

Amplified PCR products were purified and cloned into a pGEM®‐T Easy Vector (Promega KK, Tokyo, Japan). Plasmid DNA was isolated using the alkaline SDS method. Isolated plasmid DNA was sequenced using an ABI PRISM 310 Genetic Analyzer (Applied Biosystems, Foster City, CA). At least 10 clones per fetus were prepared using three independent fetuses (n = 3). As described in a previous report,^26^ we compared the percentages of methylated cytosines among all detected cytosines between controls and fetuses derived from 24 h‐oocytes.

### 2.6 Statistical analyses

Statistical analysis of data for developmental rates, body weight, and feed consumption was performed using one‐way analysis of variance and Student's *t* test using Statview software (Abacus Concepts, Inc, Berkeley, CA). A *P* value <0.05 was considered statistically significant.

## 3 RESULTS

### 3.1 Inchmeal decline of developmental competence into the blastocyst stage in embryos derived from postovulatory oocytes

**Figure 1:**
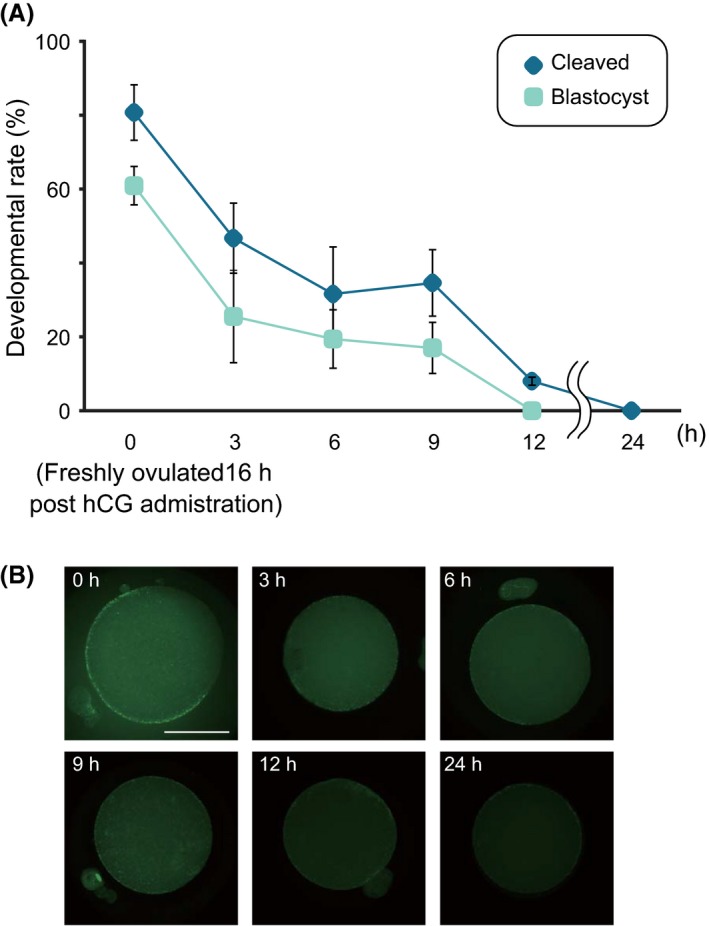
Deterioration of metaphase of the second meiotic division (MII) oocytes after postovulatory incubation. (A) The rates of cleaved embryos (dark blue) and blastocysts (light blue) among all cultured embryos are shown. As the duration of incubation after oocyte retrieval from super‐ovulated females increased, both cleavage rates decreased (0 h, n = 157; 3 h, n = 137; 6 h, n = 155; 9 h, n = 153; 12 h, n = 175; and 24 h, n = 77). (B) The effect of postovulatory aging on the dynamics of cortical granules (CGs) using Alexa Fluor 488‐conjugated lectin PNA. The distribution of CGs is usually considered one of the most important indicators of oocyte cytoplasmic maturation. Note that the fluorescent signals for CGs under the oocyte subcortex were extensively decayed in 12 h‐ and 24 h‐oocytes compared with those in 0 h‐oocytes. More than 20 oocytes were evaluated at each time point. Bar = 50 μm

We first confirmed that the IVF embryos derived from postovulatory oocytes exhibited decreased cleavage and subsequent development to the blastocyst stage depending on the duration after retrieval from oviducts (Figure 1A). The rate of cleaved embryos was first assessed at 24 h after IVF. In the 6 h‐ and 9 h‐oocytes, the cleavage rates decreased to less than 50%, concomitant with the decrease of blastocyst formation rates to approximately 20% (Figure 1A). Particularly, only 8.0% of the embryos derived from the 12 h‐oocytes were cleaved, and none developed to the blastocyst stage. In addition, none of the embryos derived from the 24 h‐oocytes were cleaved. These inchmeal degradations of oocytes obviously depended on the postovulatory duration, which was supported by the gradual depression of fluorescent intensity for oocyte CGs, causing hardening of ZP (Figure 1B).

### 3.2 Developmental competence to term in embryos derived from oocytes senesced after ovulation

**Figure 2:**
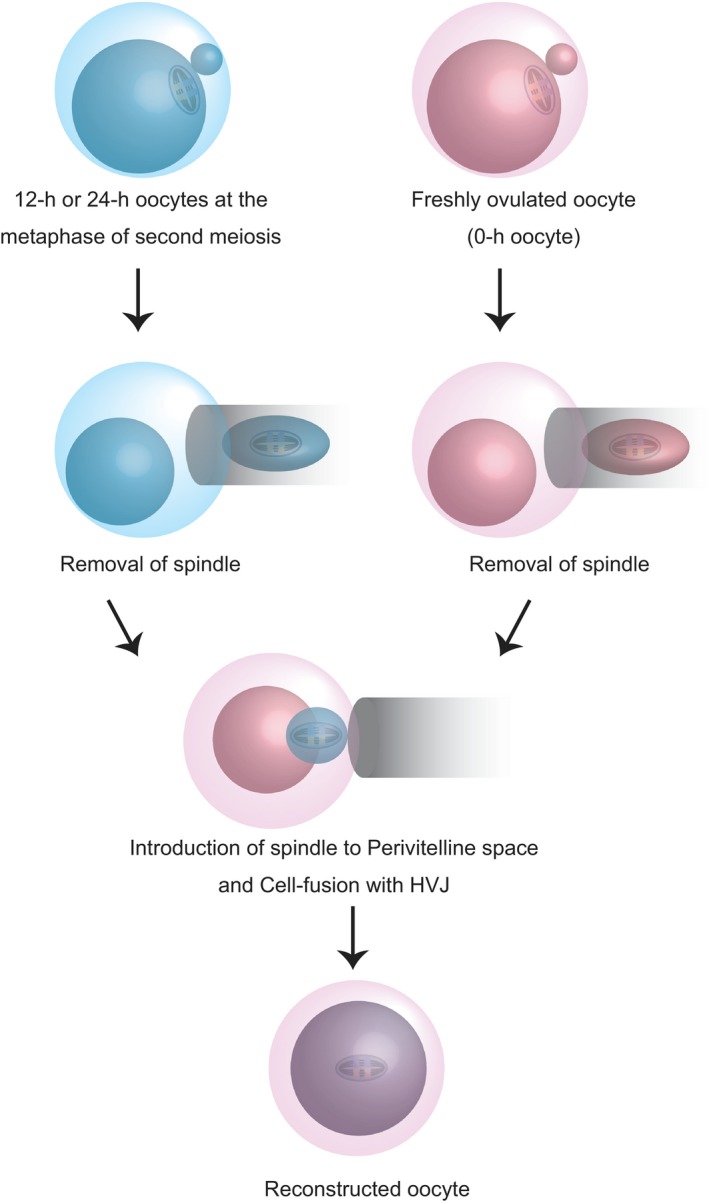
Schematic representation of oocyte spindle transfer to produce mice from postovulatory‐aged oocytes after 12 h or 24 h of incubation

**Figure 3:**
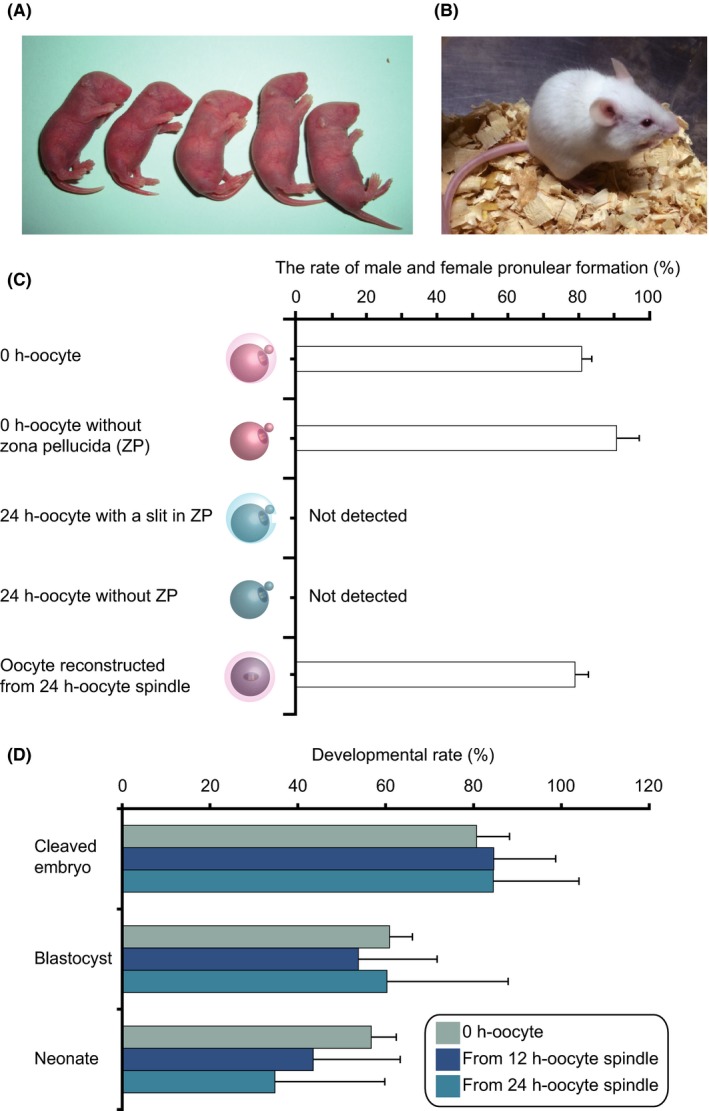
Developmental competence of oocyte spindle transfer (OST)‐derived embryos. (A) Five neonates produced from oocytes that were reconstructed using 24 h‐oocyte spindles. (B) A 4‐week mouse that was produced via OST using a 24 h‐oocyte spindle. (C) The rate of male and female pronuclear formation in five types of presumptive zygotes: using 0 h‐oocytes (n = 99), 0 h‐oocytes without zona pellucida (ZP) (n = 43), 24 h‐oocytes with a slit in ZP (n = 55), 24 h‐oocytes without ZP (n = 48), and OST‐oocytes reconstructed from 24 h‐oocyte spindles (n = 114). (D) Developmental rates of OST‐derived embryos to the neonatal stage (0 h‐oocytes, n = 126; from 12 h‐oocyte spindles, n = 121; from 24 h‐oocyte spindles, n = 49). The data represent the rates of cleaved embryos (0 h‐oocytes, 80.7 ± 7.5; 12 h‐oocyte spindle, 84.6 ± 14.1; 24 h‐oocyte spindle, 84.5 ± 19.5), blastocysts (0 h‐oocytes, 60.9 ± 5.2; 12 h‐oocyte spindle, 53.8 ± 17.9; 24 h‐oocyte spindle, 60.3 ± 27.6), and neonates (0 h‐oocytes, 56.7 ± 5.7; 12 h‐oocyte spindle, 43.5 ± 19.5; 24 h‐oocyte spindle, 34.8 ± 25.0). Non‐manipulated controls, blue gray; OST‐derived embryos using 12 h‐oocyte spindles, dark blue; and OST‐derived embryos using 24 h‐oocyte spindles, sky blue

According to the results for the inchmeal degradation of postovulatory oocytes, we transferred MII spindles from 12 h‐ or 24 h‐oocytes into enucleated 0 h‐oocytes to restore the developmentally compromised postovulatory‐aged oocytes (Figure 2). After embryo transfer to pseudopregnant females, pups derived from either 12 h‐ or 24 h‐oocyte spindles were generated, after which they grew to adulthood (Figure 3A,B). None of the presumptive zygotes using 24 h‐oocytes with or without ZP formed the male and female pronuclear formation, although 80.7% of those from OST‐oocytes using 24 h‐oocyte spindles were fertilized with two pronuclei (Figure 3C). This result clearly showed that the deteriorated developmental quality in 24 h‐oocytes was not only due to the hardening of ZP but also due to the degradation of ooplasm.

The cleavage and blastocyst formation rates in the embryos reconstructed from 12 h‐ and 24 h‐oocyte spindles were compared with controls comprising IVF embryos generated using 0 h‐oocytes (Figure 3D). There were no significant differences in either rate between controls and embryos reconstructed from 12 h‐ or 24 h‐oocyte spindles. Furthermore, in addition to preimplantation development to the blastocyst stage, we also determined neonate production rates after embryo transfer for embryos derived from 12 h‐ and 24 h‐oocyte spindles. As expected, the embryos derived from 12 h‐ and 24 h‐oocyte spindles exhibited similar frequent development to the neonatal stage as observed for non‐manipulated controls. Although there were no significant differences in developmental rates to the neonatal stage between among control and postovulatory‐aged oocytes, the neonate production rates tended to decrease depending on the duration of incubation for postovulatory aging treatment (non‐manipulated control: neonate number/transferred embryos = 17/30, 56.7 ± 5.7%; 12 h‐oocytes, 10/23, 43.5 ± 19.8%; and 24 h‐oocytes, 8/23, 34.8 ± 25%). Taken together, these results clearly demonstrated that ooplasm exchange between postovulatory‐aged oocytes and freshly ovulated oocytes remarkably improved developmental competence for both preimplantation development and neonatal development after implantation.

### 3.3 Body weights of fetuses derived from oocytes senesced after ovulation

**Figure 4:**
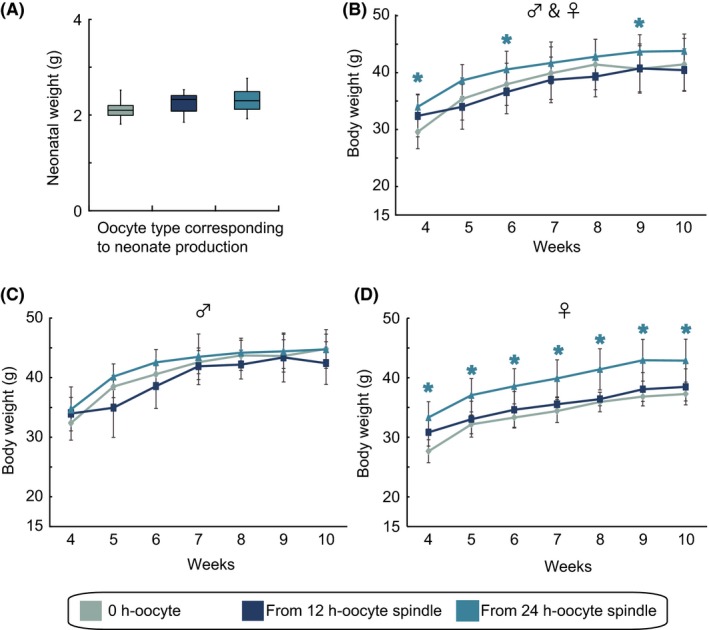
Growth of mice derived from postovulatory‐aged oocytes. (A) The body weights of neonates produced from non‐manipulated controls (n = 10) and oocyte spindle transfer (OST)‐derived embryos using 12 h‐ (n = 8) and 24 h‐ (n = 7) oocyte spindles. (B) The data represent the mean body weights ± SEM from weeks 4 to 10 in both sexes (0 h‐oocyte controls, n = 10; 12 h‐OST, n = 8; and 24 h‐OST, n = 6). The same numbers of mice of each sex were used in each group. Among the data shown in (B), the body weights of males (C) and females (D) are plotted. The same numbers of mice were analyzed for each sex. Non‐manipulated controls, blue gray; OST‐derived embryos using 12 h‐oocyte spindles, dark blue; and OST‐derived embryos using 24 h‐oocyte spindles, sky blue. An asterisk denotes statistical significance in OST‐derived mice using 24 h‐oocyte spindles versus controls (*P* < 0.05)

We investigated the growth of pups derived from postovulatory‐aged oocyte spindles. First, we determined the neonatal body weight of pups derived from postovulatory‐aged oocyte spindles. The neonates derived from the 12 h‐ and 24 h‐oocyte spindles had similar neonatal body weights as those derived from non‐manipulated IVF controls from 0 h‐oocytes (control: 2.13 ± 0.07 g; 12 h‐oocyte: 2.24 ± 0.09 g; and 24 h‐oocyte: 2.32 ± 0.07 g) (Figure 4A). Conversely, mice derived from 24 h‐oocyte spindles had significantly higher body weights at 4, 6, and 9 weeks of age than controls, although no differences were observed between controls and mice derived from 12 h‐oocyte spindles (Figure 4B). To further understand the cause of the increase in body weight in the pups derived from 24 h‐oocyte spindles, body weight was analyzed in both males and females (Figure 4C,D). Among males, there were no significant differences in body weights throughout the examined term, whereas, among females, mice derived from 24 h‐oocyte spindles had significantly higher body weights than that of controls from 4 to 10 weeks after birth (*P* < 0.05) (Figure 4D). Thus, these results revealed that the body weights of mice derived from 12 h‐oocyte spindles were equivalent to those of controls, whereas mice derived from 24 h‐oocyte spindles, particularly females, displayed significant overgrowth.

### 3.4 Feed consumption of fetuses derived from oocytes senesced after ovulation

**Figure 5:**
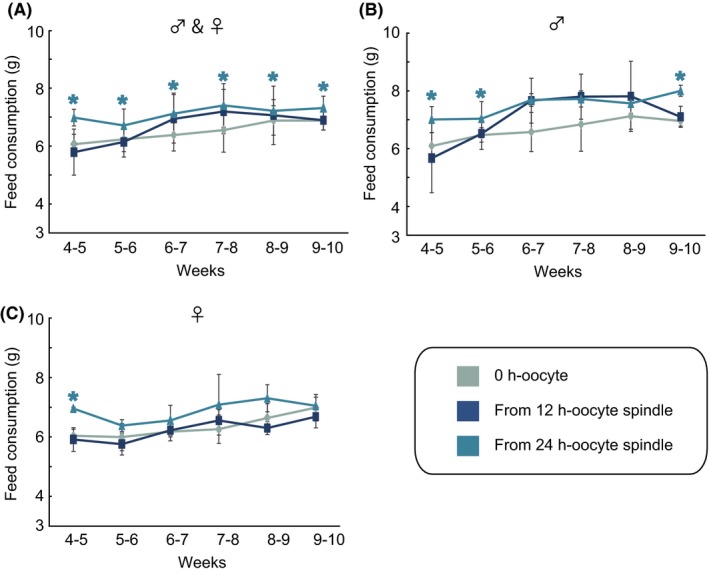
Feed consumption of mice derived from postovulatory‐aged oocytes. (A) The data represent the mean values ± SEM of feed consumption per week in neonates produced from non‐manipulated controls and oocyte spindle transfer (OST)‐derived embryos using 12 h‐ and 24 h‐oocyte spindles (0 h‐oocyte controls, n = 10; 12 h‐OST, n = 8; and 24 h‐OST, n = 6). The same numbers of mice of each sex were used in each group. Among the data shown in (A), the mean feed consumption was separately plotted for males (B) and females (C). The same numbers of mice were analyzed for each sex. Non‐manipulated controls, blue gray; OST‐derived embryos using 12 h‐oocyte spindles, dark blue; and OST‐derived embryos using 24 h‐oocyte spindles, sky blue. An asterisk denotes statistical significance in OST‐derived mice using 24 h‐oocyte spindles versus controls (*P* < 0.05)

To explore the reason why mice derived from 24 h‐oocyte spindles exhibited overgrowth, we investigated the feed consumption of the mice from 4 to 10 weeks after birth (Figure 5). In all generated individuals regardless of sex, mice derived from 24 h‐oocyte spindles, but not those derived from 24 h‐oocytes, displayed significantly greater feed consumption than controls (Figure 5A). In males, the mice derived from 24 h‐oocyte spindles exhibited significantly greater feed consumption than controls at 4‐5, 5‐6, and 9‐10 weeks (Figure 5B). In females, there was significant differences between controls and mice derived from 24 h‐oocyte spindles only at 4‐5 weeks (Figure 5C), although the feed consumption of mice derived from 24 h‐oocyte spindles tended to be higher than that of controls. Therefore, these results suggested that increased feed consumption was one cause of overgrowth among mice derived from 24 h‐oocyte spindles, indicating the irreversible failure in postovulatory‐aged oocytes after 24 h of incubation.

### 3.5 DNA methylation statuses at the *Snrpn*‐DMR in three organs of E16.5 fetuses derived from postovulatory‐aged oocytes

**Figure 6:**
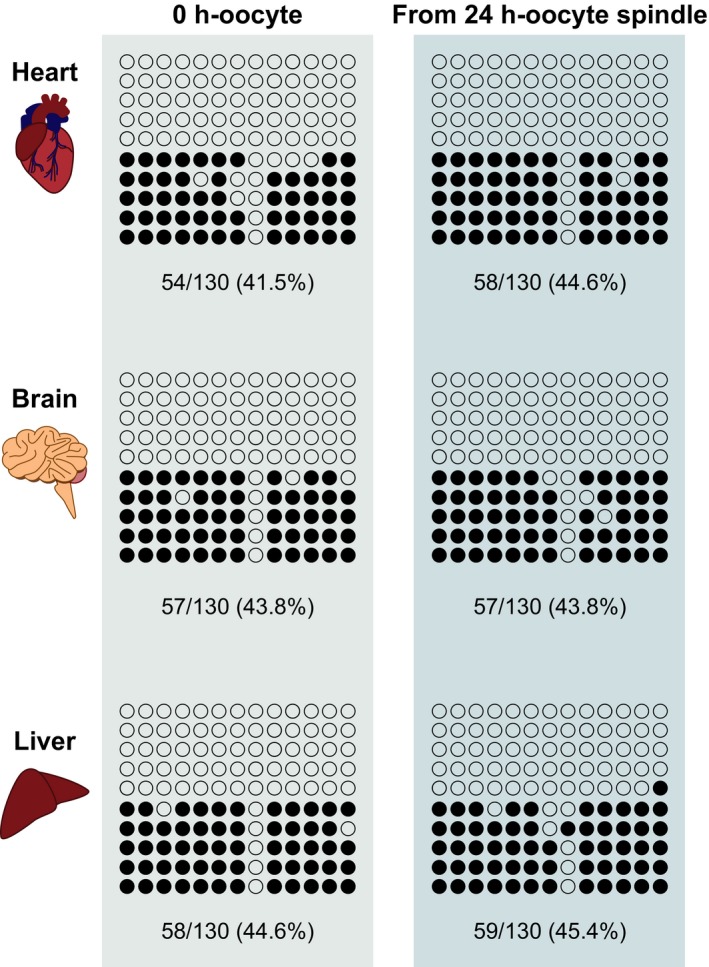
Bisulfite sequencing analysis of methylation status of the *Snrpn*‐differentially methylated region (DMR) in fetuses derived from postovulatory‐aged oocytes. CpG methylation profile of the *Snrpn*‐DMR in the heart, brain, and liver tissue obtained from controls and OST‐derived fetuses using postovulatory‐aged oocytes (n = 3). Methylated and unmethylated CpG sites are denoted by filled and open circles, respectively. The percentages of methylated cytosines among all detected cytosines were compared between control and 24 h‐oocyte‐derived mouse organs

To further explore the irreversible failure in postovulatory‐aged oocyte spindles, we assessed the methylation status at the *Snrpn*‐DMR in three primary organs, namely the heart, brain, and liver, retrieved from female fetuses at E16.5 because it was reported that the *Snrpn*‐DMR was demethylated in the postovulatory‐aged oocyte genome.^7^ We compared the percentages of methylated cytosines among all detected cytosines between the controls derived from 0 h‐oocyte spindles and fetuses derived from 24 h‐oocyte spindles. The *Snrpn*‐DMR methylation statuses of controls exhibited parent‐of‐origin‐specific patterns in three organs examined (Figure 6). Likewise, the *Snrpn*‐DMR of the fetuses derived from 24 h‐oocyte spindles displayed the same methylation level as controls in these three organs (44.6% in the heart, 43.8% in the brain, and 45.4% in the liver) (Figure 6). Thus, in the *Snrpn*‐DMR of survived fetuses derived from 24 h‐oocyte spindles, the methylation status was maintained at the same level as controls.

## 4 DISCUSSION

We first confirmed that embryos derived from 12 h‐oocytes failed to develop to the blastocyst stage, and further, none of the embryos derived from 24 h‐oocytes could cleave. Although earlier studies did not define the precise timing at which the ability to fertilize and develop were lost in postovulatory oocytes in vitro, our results were largely consistent with previous studies demonstrating the developmental failures of postovulatory‐aged oocytes.^9^, ^10^ As the fertilizable competence of postovulatory oocytes also ranges from 12 to 24 h in vivo,^27^ our results were logical in terms of the duration without fertilization. The OST technique allowed those deteriorated oocytes to fertilize and develop to term at high rates, even using 24 h‐oocyte spindles. In the present study, the rate of development to term from the reconstructed oocytes using the 24 h‐oocyte spindles was higher than that in previous reports (5/116; 4.3%,^9^ 12/141; 8.5%^11^). This does not appear to be attributable to the differences of the mouse strains used for producing reconstructed oocytes because F1 females were used in both these previous reports and oocytes derived from F1 generally possess higher quality of development in vitro and in vivo.^28^ OST led to significant improvements in ooplasm and ZP denatured by the cortical reaction of CGs, which enabled fertilization and ontogeny using postovulatory‐aged oocyte spindles.

The finding of greater overgrowth among females could be due to sex differences in energy metabolism, which is well known in humans and rodent models.^29^ The sex asymmetry in energy metabolism could have arisen from a variety of causes, including differences in adipose tissue storage,^30^ glucose homeostasis,^31^ gonadal sex hormones,^32^ and sex chromosomes.^33^, ^34^ Thus, it is extremely difficult to interpret why the overgrowth phenotype was visible only in females; however, in any rodent model, females frequently exhibited overgrowth in the aforementioned studies. Collectively, the overgrowth phenotype and binge‐eating disorder resulted in irreversible damage to oocyte spindle during postovulatory aging, indicating that transferring excessively aged oocyte spindles to the cytoplasm derived from freshly ovulated oocytes could not inspire the developmental competence needed to ensure normal postnatal growth.

Epigenetic status was assessed in this study because DNA methylation is the most widely reported epigenetic marker in postovulatory aged mouse oocytes.^7^ In postovulatory‐aged oocytes, the *Snrpn*‐DMR displayed demethylation at 29 h after hCG injection^7^ as opposed to the presence of full methylation in the same regions of normal fully grown oocytes.^35^ In normal fetuses, the methylation levels could be assumed to be 50% because of the fully methylated maternal allele and hypomethylated paternal allele.^26^ Because fetuses derived from 24 h‐oocyte spindles had similar methylation statuses as controls, the irreversible defect in excessively aged oocyte genomes is unlikely to be due to the methylation status of the *Snrpn*‐DMR.

The use of human oocytes and embryos for experiments is strictly restricted because of many complicated reasons, including ethical aspects, material availability, and the longer time required to evaluate physiological events. For these reasons, it is difficult to evaluate the feasibility of OST for rescuing deteriorated oocytes. Further, available data are also limited in mice at present to evaluate the feasibility of OST as a reproductive technology. In fact, many studies on OST have only reached the point of assessments at the cellular level, although several reports addressed the rate of development to term in mice.^9^, ^11^, ^36^ However, without assessments of postnatal development, including growth and feed consumption, it is impossible to discuss the application of OST as a practical reproductive technology. In the absence of adequate evidence regarding the wholesomeness of OST individuals, the application of this technique in human reproduction would be hazardous. The present research provides novel insights into the feasibility of OST as a potent reproductive technology and provides a warning for using excessively aged postovulatory oocytes in mammalian reproduction.

## CONFLICT OF INTEREST

The authors declare that they have no conflicts of interest with the contents of this article.

## AUTHOR CONTRIBUTIONS

MK conceived the project and designed the experiments. KS, MK, and FY performed the experiments. MK, KS, MK, and HB analyzed the data. MT contributed to conception of the work. MK, KS, MK, and HB wrote the manuscript. All the authors contributed to the interpretation of the data, and read and approved the final manuscript. Authors declare no competing interests.
